# Integrating planetary health into medical training: Defining physicians’ roles in Malawi

**DOI:** 10.4102/phcfm.v17i1.4962

**Published:** 2025-10-20

**Authors:** Arne M. Beguin, Andrea van Acker-Koning, Prosper Lutala, Martha Makwero, Dorothee van Breevoort

**Affiliations:** 1 Department of Family Medicine, Faculty of Medicine, Kamuzu University of Health Sciences, Mangochi, Malawi; 2 Department of Family Medicine, Faculty of Medicine, Kamuzu University of Health Sciences, Blantyre, Malawi

**Keywords:** planetary health, medical education, low-income setting, role of medical doctors, physician roles

## Abstract

**Background:**

Planetary health (PH) is an emerging field gaining attention in medical education. However, the specific roles of physicians in low- and middle-income countries (LMICs) in addressing PH issues are not well defined.

**Aim:**

This study aims to identify the potential roles of physicians in PH in LMICs in order to design specific teaching content for medical doctors in these regions. Moreover, this study aims to explore student baseline knowledge and student learning priorities in LMIC settings to further tailor content of teaching material within the medical curriculum.

**Setting:**

This study was conducted in Mangochi District in Malawi, a LMIC.

**Methods:**

A cross-sectional convergent mixed methods study design, including seven in-depth interviews with district stakeholders and two focus group discussions (FGDs) with medical students explored the roles of medical doctors in PH. A survey assessed students’ baseline knowledge and learning needs.

**Results:**

Four main roles of physicians in LMICs within PH were identified: healthcare provider, hospital manager, community manager and advocate. While 61% of the students had never heard of PH, 82% supported its inclusion in the curriculum. Priority areas to be included in the curriculum were extreme weather events, links between PH and non-communicable diseases (NCDs), vector-borne diseases and mental health.

**Conclusion:**

Stakeholders identified essential roles for physicians to address challenges in relation to PH.

**Contribution:**

Key roles of physicians within planetary health, and medical students’ learning priorities were identified, and could aid in designing teaching material for medical students in LMICs.

## Introduction

Planetary health (PH) can be defined as the health of human civilisation and the state of the natural systems on which it depends.^1^ It arose as a prominent concept in 2013 when Horton et al. identified the idea that ‘global health’ should be extended towards the planet and not focus solely on health issues and solutions.^2^ This extension is needed, as most of the progress of healthcare in the last few decades, shown by increased life expectancy and reduction of maternal and child mortality, has been achieved by exploiting natural resources.^1^ Currently, these acquired health benefits are increasingly challenged by the adverse impacts of this exploitation through climatic change, ocean acidification, land degradation, water scarcity, overexploitation of fisheries and biodiversity loss.^3^ These adverse impacts can result in increased malnutrition, changing habitats for vectors of disease, increased prevalence of non-communicable diseases (NCD), displacement and conflict, and a rise in mental health conditions.^1^ Planetary health sets the ambitious task of understanding the dynamic and systemic relationships between global environmental changes, their effects on natural systems and how changes to natural systems affect human health and wellbeing at multiple scales: global, regional and local. By emphasising interconnections between human health and environmental changes PH enables holistic thinking about overlapping challenges and integrated solutions for present and future generations.^4^

Embedding PH education in medical curricula is an essential step for healthcare professionals to understand these complex interactions^1,5^ and to be enabled and equipped to drive the transformative systems change needed to protect and restore PH and achieve the Sustainable Development Goals (SDGs).^6^ Incorporation of PH in medical institutions has already started in some countries. A survey from 2020 showed that 15% of a total of 2817 medical schools in 112 countries had already incorporated PH in their curriculum,^7^ although sub-Saharan Africa was underrepresented in the study. Primary health care leaders in sub-Saharan Africa have emphasised the need to strengthen PH capacity in the region.^8^ In alignment with this, a 2023 position statement published in the *African Journal of Primary Health Care and Family Medicine* called for the integration of PH and environmental sustainability into the training of health professionals across the continent.^9^

A generic PH education framework has been established by a taskforce of leaders in PH and education to facilitate the transformation of curricula towards PH inclusion.^10^ Tailoring this framework to the learners’ setting and focussing on local learning needs, will be most efficient and effective. Currently, most PH curriculum integration and PH short courses to teach PH are in institutions in high-income settings.^11^ Similarly, most expert opinions about the physician’s role in the Anthropocene era, in the context of PH have been published by researchers in high-income settings as well.^12,13,14,15^ To our knowledge, limited research has been conducted to understand the specific roles of physicians from low- and middle-income countries (LMICs) in relation to PH and no structured multisectoral assessment been undertaken to investigate the physician’s role in LMICs in this domain.

This study aims to identify the potential roles of physicians in LMICs in relation to PH – identifying the crisis of the Anthropocene era, formulating accurate prognosis and proposing solutions – in order to design specific teaching content for medical doctors in these regions. Moreover, this study aims to explore student’s baseline knowledge and student learning priorities in LMIC settings to further tailor content of teaching material within the medical curriculum.

## Research methods and design

### Study design

This study used a cross-sectional convergent mixed methods study design, including in-depth interviews with district stakeholders from multiple sectors, focus group discussions (FGDs) with medical students at a district family medicine rotation and a survey among all 4th year medical students at the only university of health sciences in Malawi.

### Setting

The study was conducted in Mangochi District, a rural district in eastern Malawi with a population of 1.1 million people.^16^ The district stakeholders who were interviewed worked in Mangochi District. Medical students included in the study are students at the Kamuzu University of Health Sciences in Blantyre, Malawi; the only medical university in Malawi. The medical curriculum, a medical bachelor and bachelor of surgery (MBBS), is a 5-year curriculum, including 3 years of theory and 2 years of internship. The FGDs were conducted in the fourth-year of the programme during the 6 weeks of the students’ family medicine district hospital rotation in Mangochi.

### Selection of participants

For the in-depth interviews, participants were purposely selected among key district officials representing multiple sectors involved in PH, specifically; health, forestry, environment, agriculture and emergency response. All participants were recruited from a single, purposively selected district in Malawi – Mangochi district – as it hosts the only rural satellite site affiliated with the Department of Family Medicine. Inclusion of participants continued until saturation was achieved, that is, the point at which no new themes, insights or perspectives were emerging from additional interviews.

For the survey, all 4th-year medical students at the medical university in Malawi were approached after taking the end of rotation exam in August 2023 and invited to participate. All 4th-year medical students who were randomly assigned to conduct the family medicine rotation in Mangochi, were invited by the principal investigator to participate in the FGDs, during their third week in the rotation after an afternoon teaching session.

### Data collection

A semi-structured interview guide was developed for the in-depth interviews to explore the perceptions among the district leadership on the physician’s role in the context of PH. The interview guide was developed using the Planetary Health Education Framework and additional relevant literature, and aligned with study objectives.^10,17,18^ The in-depth interviews were conducted face-to-face in the English language and audio recorded for subsequent analysis.

Two FGDs were carried out to explore the students’ pre-knowledge about PH and to explore their perception towards the role of physicians in PH. A topic guide was created based on the Planetary Health Education Framework.^10^ Two FGDs were conducted in-person in English, both lasting between 45 min and 1 h, and both were audio recorded.

Survey questions to investigate students’ pre-knowledge, interest and learning priorities in PH were adopted from a validated survey used in a similar study in Germany, after being adjusted for the local setting to align with the local medical curriculum and using local terminology.^18^ The survey was adapted to incorporate all components of the PH education framework from Guzmán et al. that were missing; it is available in Online Appendix 1.^10^ Students were asked during the survey to select a maximum of five topics within PH that captured their greatest interest.

The sample size for the survey to assess background knowledge and learning priorities was calculated to be 92, based on an assumed proportion of 50% for awareness of PH among medical students, with a 95% confidence level and a 5% margin of error. In order to obtain at least 92 participants, the survey was shared with all 120 fourth-year medical students from the Kamuzu University of Health Science, the only medical university of Malawi. The message contained background information and an URL to the survey. Everyone was reminded on day 5, and after day 10 the URL expired. The survey was anonymous, and it was made clear that if students did not take part in the survey, it would not have any consequences for their grading or influence their study process in any way.

### Data analysis

An inductive thematic approach was used for qualitative data analysis by the principal investigator. The in-depth interviews and the FGDs were transcribed *verbatim* and significant statements related to the study questions were extracted from the transcripts. From these statements, meanings were formulated by two independent researchers and these meanings were clustered according to coding themes by both researchers. The analyses of both investigators were compared and integrated, and validated by a third investigator, who read three transcripts and identified the themes to ensure reliability of the coding. The survey data were analysed in a descriptive manner using Statistical Package for the Social Sciences (SPSS) to determine the frequency of responders.

### Ethical considerations

Ethical approval to conduct this study was obtained from the College of Medicine Research and Ethics Committee (No. P.05/23/4102). All participants signed an informed consent form prior to participating, and participants were able to leave the study at any time without any consequences. In all arms of the study, participants were assigned a code to protect their identities.

## Results

### Participant characteristics

Firstly, we interviewed seven participants, three from the health sector, one from the agricultural sector, one from the forestry department and two from the district council. All participants are residing in Mangochi semi-urban area. Participant characteristics are listed in Table 1. Secondly, 14 4th-year medical students on the family medicine rotation were invited to participate in two FGDs, and all agreed to take part. The FGDs were conducted with seven participants each, ensuring an equal distribution of male and female students. Finally, a survey was conducted among 4th-year medical students at Kamuzu University of Health Sciences. All 120 students were invited to participate, 93 responded (78% response rate), including 59 males and 34 females, aged between 23 years and 35 years.

**TABLE 1:** Study participants’ characteristics.

Variable	In-depth interview	Focus group discussion	Survey	
			*n*	%
**Sex**				
Male	4	5	59	37
Female	3	9	34	63
Age range (years)	24–60	23–28	22–35	-
**Sector**				
Health	3	14	93	100
Agriculture	1	-	-	-
Forestry	1	-	-	-
District Council	2	-	-	-

### Potential roles of medical doctors in planetary health

**FIGURE 1:**
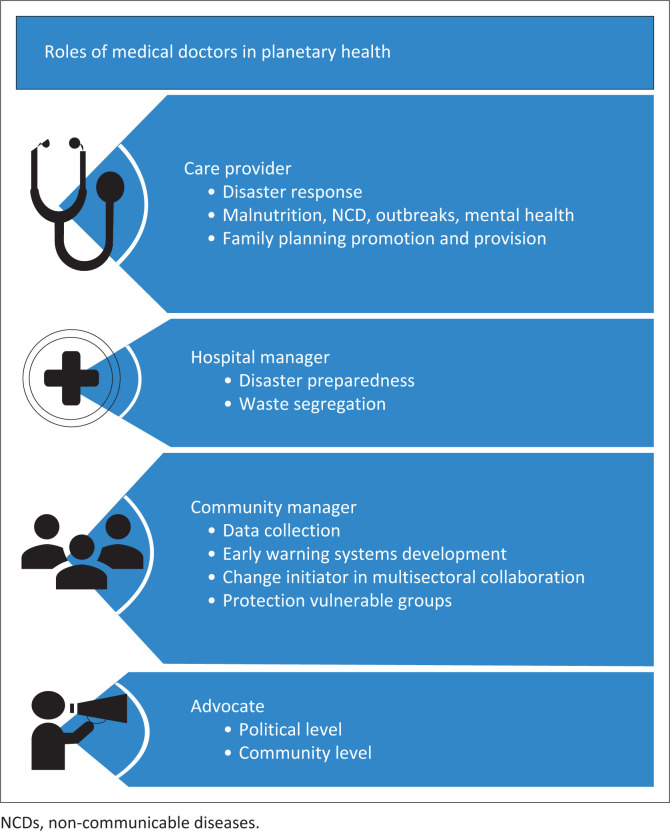
Roles of medical doctors in planetary health according to district stakeholders and medical students in Malawi.

Regarding the potential roles of physicians in the PH context, four main themes emerged from the in-depth interviews with stakeholders and the FGDs with medical students. These themes are: (1) healthcare provider; (2) hospital manager; (3) community manager; and (4) advocates of PH (Figure 1).

#### Theme 1: Healthcare provider

First of all, the role of providing medical care was mentioned by several stakeholders and students as one of the core tasks of physicians. Providing direct medical patient care or supervising care given to victims of climate-related disasters, such as flooding, droughts and outbreaks, is a key responsibility of a medical doctor, according to the participants. Furthermore, it was mentioned that promotion and improvement of family planning provision is also a primary duty of physicians. The latter will directly reduce further population growth and subsequently the need for natural resources. Moreover, increased use of family planning methods will indirectly reduce poverty and subsequently have a positive effect on deforestation for farmland, construction and charcoal production:

‘Population to grow like this, is the major cause of the problem [*of climate change*]. We need to intensify the education about family planning methods to women of child bearing age and their husbands. Medical doctors are the ones finally responsible for this.’ (Female 45 years, medical sector, IDI)

‘Doctors are responsible for emergency response in case of emergencies, but also for things like stimulation of sanitation around the house, hand hygiene, family planning and child spacing all those things help to make a difference.’ (Female 24 years, student)

#### Theme 2: Hospital manager

A second role for physicians that was mentioned is the role of hospital manager. Physicians are responsible for health facilities to have disaster preparedness plans to improve the resilience of the facility to climate-related disasters, including cyclones, floods and famines. Recognition of (climate-related) outbreaks, by monitoring patient categories and conditions, was also mentioned. Moreover, the hospital’s own contribution towards PH problems should be considered and addressed by physicians as being overall responsible for the facility. Improving waste management, reduction of the use of disposables and change towards solar-generated power instead of generators, were some of the examples mentioned:

‘We are contributing a lot to the issue [*of climate change and pollution*] for example the gloves and cotton that we use. How we dispose them, plays a very big role. So, we may think of introducing more of reusable equipment for example the specula; we can use metal ones because it can be cleaned and be used again and so we can stop using the plastic ones.’ (Male 25 years, student, FGD)

#### Theme 3: Community manager

Participants identified the role of addressing PH challenges for physicians within district management. Even though physicians are almost exclusively trained in biomedical knowledge, it was mentioned by the participants that they end up taking a major role in district management. They function, for example, as district medical officers or directors of health and social services. Firstly, it was mentioned that in the district, doctors have the opportunity to collaborate with other sectors to improve data collection for advocacy, research and development of early warning systems. Secondly, developing district emergency plans was brought up as part of the tasks of physicians in the district. Thirdly, it was stated that physicians could serve as change initiators within the district management teams or even step up as leader of the other sectors towards efforts to mitigate health effects of PH challenges. The first example mentioned was increasing efforts within district management to initiate strategically placed reforestation projects to prevent mudslides and collapsing infrastructure. Another strategy mentioned by several participants was stimulation of district initiatives towards climate-resilient agriculture methods and crop diversification to prevent malnutrition and unnecessary deforestation for additional agricultural fields. A final example mentioned was boosting the district leadership commitment towards discouraging the use of wood-based cooking methods, among others, by penalising charcoal production or investing in alternatives, such as biogas or fuel-efficient stoves. A last role of medical doctors as community managers, according to participants, is their responsibility to put mechanisms in place to ensure protection of vulnerable groups from climate-related impacts, such as pregnant women, children and the elderly:

‘They [*physicians*] can even take the lead if things are not working in the district.’ (Female 47 years, environmental sector)

‘Medical doctors are responsible as lead of the health sector to make sure outreaches are organised to reach the vulnerable people, during floods for example.’ (Female 45 years, medical sector)

#### Theme 4: Advocates

In addition, the role of physicians as advocates was addressed by the respondents. Advocacy towards government at national level was mentioned by presenting climate-related health data in order to demand a change in legislation and regulation. Examples mentioned were banning of old vehicles, regulations about pesticides and stimulation of renewable energy production. Also, advocacy at the community level was brought up. It was mentioned that medical doctors are in a better position than some other sectors to take up this role as advocates in the community, because people have trust in doctors. Examples of measures physicians could advocate for are measures to mitigate effects of climate change related disasters, such as relocating from flood prone areas, ensuring afforestation of hills surrounding villages or promoting compost use instead of chemical fertiliser:

‘Medical doctors are in a better position than some other sectors to take up this role as advocate towards the community, because people have a higher trust in doctors then politicians.’ (Female 64 years, forest sector)

‘It means I have to be an advocate for forestation. It means I have to be an advocate for a good farming method, maybe relocation of people to less flood prone areas, things like those. But if I’m only concentrating on health issues, then I’m not doing justice.’ (Male 24 years, healthcare sector).

### Pre-knowledge of planetary health and interest to include the subject in the curriculum

In all, 65% (*n* = 60) of the 93 medical students who filled the survey indicated that they have never heard about PH before; even though after explaining the concept, 82% (*n* = 76) of the students stated that it is important or rather important to implement PH in the curriculum. The majority (*n* = 72, 74%) indicated that 1 week or longer should be allocated within the medical curriculum for this topic. Even if PH were not included, 84% (*n* = 78) of the participants reported that they would likely choose an online course about PH if it were available.

### Learning priorities about planetary health from medical students

**FIGURE 2:**
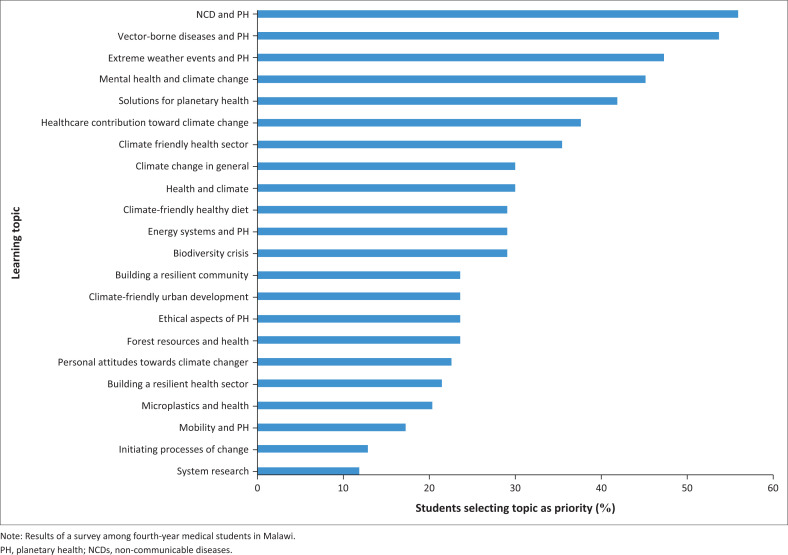
Priority areas of planetary health for the medical curriculum according to medical students (*N* = 93).

The greatest topic of interest among the medical students was NCDs. Other major interests of the students were vector-borne diseases, extreme weather events and mental health. The results are outlined in Figure 2.

## Discussion

This study identified four potential key roles of physicians in the crisis of the Anthropocene era in the context of PH within rural districts in Malawi, a low-income country. Apart from the traditional role as health care provider, physicians in low-income countries can play major roles in hospital management, district health management and advocacy at both national and community levels. Physicians have the opportunity to limit the use of unnecessary resources, and to reduce pollution from the health sector directly, by improving waste segregation and waste handling. More importantly, however, they could function as change initiators or even leaders within district management to increase the resilience of the health sector and the district population to the effects of climate change. Despite these identified potential roles for physicians, PH is not part of the medical curriculum in Malawi. Nonetheless, medical students are interested in the subject; the majority in this study stated that they would even be willing to join extracurricular online courses about PH.

The identified roles align with findings in the literature; The Planetary Health Alliance, a group of 300 organisations from over 60 countries, defines roles of physicians in relation to PH as being responsible to recognise climate change related patterns of diseases, to manage green health care practices and to serve as entrusted messengers, community builders and educators.^19^ Expert opinions from Australia and Slovenia identified additional roles as leaders, change initiators, direct healthcare providers in climate-related emergencies and protectors of the vulnerable.^20,21^ In sub-Saharan Africa, the broader role of the physicians has been described by a group of family physicians during the world organization of family physicians (WONCA), during their regional African conference as advocates towards both civil society and government, medical care providers, collaborators, educators, and researchers.^8^

In our study, we identified that physicians could function as change initiators or as leaders in these proposed roles in hospital and community settings, as well as direct healthcare providers and community advocates. The roles of educators and researchers about the concept of PH were not mentioned by any of the participants directly. The lack of these potential roles might be partially explained by the study location, a rural district where limited scientific research is carried out. Furthermore, the knowledge gap among medical students, as shown by the survey, indicates a lack of focus on PH in the medical curriculum. Without exposure to medical professionals teaching PH, it becomes more challenging for students to recognise the potential role of physicians as educators in this field.

While we focused on general roles, Ezeh et al.^22^ suggest focussing on three specific key interventions: family planning; management of natural resources, including reforestation; and regulation for urbanisation. Family planning and reforestation have been mentioned in the FGD in our study as well, under the roles of health care provider and community manager, respectively. The specific role of regulating urbanisation was not identified in this study, which may be attributed to the semi-urban context in which stakeholders were consulted – an environment where urban planning is likely less prominent. Moreover, the inclusive design of our study allowed for the identification of additional roles for physicians, including advocacy, management of both hospital and community care, as well as several other health-related functions such as disaster response, mental health services, and malnutrition care.

The four key roles identified in this study contribute to the clinical applicability of the existing theoretical *Planetary Health Education Framework*, developed in 2021 by experts in the field of medical education.^10^ This framework outlines five key domains of PH education. The advocacy role identified in our study aligns with the framework’s dedicated advocacy segment. Similarly, the roles of community manager and hospital manager correspond to domains such as systems thinking, the Anthropocene and health, and equity and social justice. However, the role of providing clinical care in the context of PH is not explicitly addressed in the framework. Given that our findings highlight the importance of adapting medical care to the evolving challenges posed by PH issues, it would be valuable to incorporate this role more explicitly into PH education.

Pre-knowledge of medical students about the concepts of climate change and PH is different from other literature. In South Africa, a recent survey among undergraduate health science students showed a higher pre-knowledge regarding the concept of climate change, with 65% of 264 students claiming to be aware of climate change.^23^ However, the concept was poorly understood and only 47% thought it was important to be incorporated in the medical curricula.^23^ In our study we found this to be 26% and 81%, respectively. This could be explained because climate change is an older concept than PH, so it might be well-known. However, the limited understanding of the terminology might decrease the interest of the students in South Africa. A study conducted in Ethiopia indicated a slightly higher awareness of the concept of climate change, with 77% among 306 health-related students being aware of the concept.^24^ In a study among 1039 nursing students in several Arabic countries the awareness was comparable, between 73% and 100% depending on the country.^25^ Willingness to add it to the curriculum was however, not assessed in these studies.

A study addressing PH specifically was conducted in Brazil, where 83% of 75 medical students defined PH education as relevant.^26^ In a German study among 1303 medical students, 82% indicated it was important to integrate PH into the medical curriculum, even though 74% of the students were not familiar with the term PH before the study.^18^ These levels of interest are comparable to the 82% in our study. In addition, PH is a matter of importance according to medical students in other settings is also shown by literature reporting student-led initiation of PH in medical curricula or student initiatives towards adding PH to medical curricula.^27,28^

An assessment of student-suggested learning priorities among 1303 German medical students showed partly comparable preferences to the priorities identified in this study: mental health and extreme weather events were also included in the top five of student priorities. Some differences were also identified, as 56% of 93 students in this study indicated that NCDs and PH would be a priority to integrate into the medical curriculum, whereas only 35% from the medical students in the high-income setting of Germany identified this as a topic of interest.^18^ Learning priorities among the links between diseases and climate change in Brazil were obesity, cardiovascular diseases and post-traumatic stress disorder.^26^

Even though these study results could aid PH curriculum development in a low-income setting, there are some limitations to this study. Firstly, this study only identifies the roles of physicians in the crisis of the Anthropocene era in the context of PH, the pre-knowledge of medical students and the learning priorities of students. It does not take into consideration all necessary steps to achieve curriculum change: strategies to add PH into existing subjects, ways to invest in faculty development, addressing of social-political dimensions or strategies to collaborate with sectoral partners.^29^

Secondly, this study focused on district healthcare because only stakeholders residing in the semi-urban setting from Mangochi town were included. They might have a different perspective towards the roles of medical doctors compared to the urban stationed stakeholders. Thirdly, only 4th-year medical students – those at the beginning of their clinical training – were surveyed. Learning priorities may vary among more advanced students in the final year of the medical programme. Moreover, the exclusive focus on medical students limits the generalisability of the findings to other health science disciplines. Finally, from a methodological standpoint, the survey instrument was adapted from a tool validated in a European context and has not been specifically validated for use in Malawi. As such, differences in cultural and educational backgrounds may have influenced students’ interpretation of the questions.

## Conclusion

Planetary health helps to identify the crisis of the Anthropocene era, makes an accurate prognosis and proposes solutions. One solution is PH education for stakeholders, such as medical doctors. Even though PH is a topic of interest among medical students in Malawi, it is not part of the current medical curriculum. In order to develop teaching material, the potential roles of physicians in this crisis have been identified at four different levels according to district stakeholders. Firstly, the provision of medical care in the changing environment influenced by the consequences of climate change. Secondly, physicians play a role in the hospital management, both by reducing the institutional contribution to climate change and in enhancing resilience to its health impacts through improved emergency preparedness. Thirdly, physicians have a role in community management by increasing intersectional collaboration to collect climate-related data collection, to organise emergency response initiatives, to protect vulnerable groups and to enhance community engagement. Finally, the role of advocate involves influencing policy at the political level and driving behavioural and structural change within communities. These roles are of crucial consideration when developing a PH curriculum in low- and middle-income settings. In addition, the main identified interest of medical students could be incorporated within the teaching to gain more interest of medical students: PH in relation to NCDs care and vector-borne diseases, mental health and health impacts of extreme weather events. These identified roles and student priorities can serve as a foundation for the development of context specific teaching material for the medical curriculum, in low-income countries where roles and responsibilities of medical doctors might differ from high-income settings.
